# Invasive Pulmonary Aspergillosis in Patients with SARS-CoV-2 Infection: A Systematic Review of the Literature

**DOI:** 10.3390/diagnostics10100807

**Published:** 2020-10-10

**Authors:** Anna Apostolopoulou, Zerelda Esquer Garrigos, Prakhar Vijayvargiya, Alexis Hope Lerner, Dimitrios Farmakiotis

**Affiliations:** 1 Division of General Internal Medicine, University of Pittsburgh Medical Center, Pittsburgh, PA 15213, USA; apostolopouloua@upmc.edu; 2 Division of Infectious Diseases, Department of Medicine, University of Mississippi Medical Center, Jackson, MS 39216, USA; zesquergarrigos@umc.edu (Z.E.G.); pvijayvargiya@umc.edu (P.V.); 3 Division of Infectious Diseases, Department of Medicine, Mayo Clinic School of Medicine, Rochester, MN 55905, USA; 4 Division of Infectious Diseases, Department of Medicine, The Warren Alpert Medical School of Brown University, Providence, RI 02903, USA; lexi_lerner@brown.edu

**Keywords:** aspergillus, aspergillosis, SARS-CoV-2, COVID-19, CAPA

## Abstract

In this systematic review, we investigate the epidemiology, pathogenesis, risk factors, clinical manifestations, diagnosis and treatment of COVID-19-associated pulmonary aspergillosis (CAPA). We identified 85 cases from 22 studies. The frequency of CAPA is currently unknown but ranges between <5% to >30% in different case series; the possibility of colonization rather than invasive disease is the most important confounder. The vast majority of patients with CAPA did not have any of the classic host risk factors, such as immunosuppression from organ transplant or neutropenia, although a significant proportion (46%) had received corticosteroids. Age, pulmonary comorbidities and male sex were associated with higher mortality. Patients treated with voriconazole had numerically lower case-fatality rate. Clinical vigilance for CAPA is advisable in critically ill patients with COVID-19 who are not improving, even those who do not meet classic host criteria for invasive mycoses, especially if they are receiving corticosteroids. A thorough, multi-faceted diagnostic work-up and early initiation of a mold-active triazole may be lifesaving. Further research studies using standardized, uniform definitions of invasive disease and colonization are urgently needed.

## 1. Introduction

According to the World Health Organization (WHO), since December 2019 and as of 26 July 2020, there have been more than 15 million documented cases of coronavirus disease 2019 (COVID-19) worldwide. Globally, more than 640,000 deaths have been attributed to the disease and its complications [1], including acute respiratory distress syndrome (ARDS) and secondary bacterial and fungal infections. According to a systematic review and meta-analysis of thirty studies (N = 3834 patients) from the earlier days of the SARS-CoV-2 pandemic (January–April 2020) [2], the rate of bacterial co-infections was 7% in hospitalized patients with COVID-19, but was as high as 14% in studies that solely included patients in the intensive care unit (ICU). The authors noted a relative paucity of data regarding COVID-19 associated fungal infections at the time. 

Since then, a rising number of cases of invasive pulmonary aspergillosis (IPA) complicating COVID-19 infections have been reported in the literature. The aim of this systematic review is to investigate the epidemiology, pathogenesis, risk factors, clinical manifestations, diagnosis, treatment and prognosis of COVID-19 associated pulmonary aspergillosis (CAPA).

## 2. Materials and Methods

We searched PUBMED for eligible studies published until July 26, 2020, without language restrictions, using the search terms (“Coronavirus/” OR “COVID/” OR “SARS/”) AND (“aspergillosis/” or “aspergillus/” or “fungal/”). We also searched the reference lists of identified articles and reviewed relevant peer-reviewed journals up to 26 July 2020. The first (AA) and senior (DF) author independently screened the abstracts of identified studies and reviewed the full texts of those that were potentially eligible; discrepancies were resolved by consensus. We excluded publications that did not report primary data (reviews), publications that reported cases of IPA associated with other viruses, or other invasive fungal infections associated with SARS-CoV-2, and cases of *Aspergillus* colonization. We excluded publications that reported isolation of *Aspergillus* from respiratory samples of patients with COVID-19, but the authors did not specify whether these represented cases of IPA or colonization, and the data provided were not enough to make that distinction. We also excluded case series with missing individual patient data regarding risk factors, diagnosis, treatment and clinical outcomes, which would hinder further analysis.

We defined patients as “immunocompromised”, if they met classic host criteria according to the European Organization for the Research and Treatment of Cancer/Mycosis Study Group (EORTC/MSG) definitions [3]. We also noted as a separate group of “immunomodulated” patients those who had some degree of immunosuppression (such as low-dose steroids, chemotherapy or hematologic malignancy) but without meeting classic host criteria. 

All cases were reviewed independently by three infectious disease attending physicians (ZEG, PV, DF), one internal medicine resident (AA), and one medical student (AHL). Data from individual studies were extracted using a predefined template. We collected data on epidemiology (age, gender, comorbidities), clinical and radiographic features, diagnostic methods, criteria used to define IPA, *Aspergillus* species, in-vitro resistance, treatment regimens and clinical outcomes. We included cases of possible, probable or proven IPA according to the EORTC/MSG criteria [3] and putative cases according to the alternative clinical algorithm proposed by Blot et al., [4]. We elected to report each case as classified by the authors. A minority of cases of IPA were not explicitly classified, for those cases we used the diagnostic data provided by the authors and classified them per EORTC/MSG criteria to the best of our ability. 

All denominators refer to number of patients with available clinical information for that specific parameter. Categorical variables were compared with x^2^ or Fisher’s exact (for expected frequencies <5) tests. Continuous variables were compared with Student’s *t*-test or the Mann–Whitney U criterion, for variables that had normal distribution (assessed by the Shapiro–Wilk test) or not, respectively. Lastly, we used R (version 3.6.3) to generate an UpSet plot for diagnostic modalities.

## 3. Cases Retrieved

A total of 85 patients with COVID-19-associated pulmonary aspergillosis (CAPA) from 22 articles were included in our analysis. Four case series were excluded, one where the authors reported isolation of *Aspergillus* from respiratory samples of patients with COVID-19, but did not specify whether these represented cases of IPA or colonization, and the data were inadequate to make that distinction [5]. Another study was excluded because of missing individual patient data [6]. The third one was an autopsy study where there was no evidence of IPA in any of the patients that met diagnostic criteria ante-mortem [7]. Lastly, we excluded the largest to-date case series of CAPA among intubated patients with COVID-19 since the authors did not provide patient-level information, with the exception of the number of patients treated with voriconazole and their mortality rates, compared to those who did not receive that treatment [8].

## 4. Epidemiology

The frequency of CAPA ranged from 35% [9] to 3.8% [10] of all ICU patients with COVID-19. In comparison, the rate of influenza-associated (model post-viral) pulmonary aspergillosis (IAPA) is estimated between 16% and 23%. The majority of CAPA cases were documented in subjects from European countries (76/85, 89.4%), with only six cases described in in Asia, two in South America, and one in Australia. In 21 out of 22 articles (*n* = 65), the authors reported the patients’ age and sex. The mean age at the time of presentation was 67 years, and most patients were male (75.4%, 49/65 with available information) (Table 1). 

With the exception of one study, the source of potential exposure to *Aspergillus* spp. was not investigated. In the study by Rutsaert et al. [9], an environmental source was ruled out by sampling room air and the oxygen and pressurized air supplies. In addition, high-efficiency particulate air filters were installed in the ICU. It is plausible that some of these cases had previous *Aspergillus* spp. airway colonization that progressed to clinical infection, since use of broad-spectrum antibiotics and especially corticosteroids, common in critically ill patients, promotes *Aspergillus* growth and tissue invasion [11,12,13,14,15,16,17,18].

## 5. Pathogenesis

**Figure 1:**
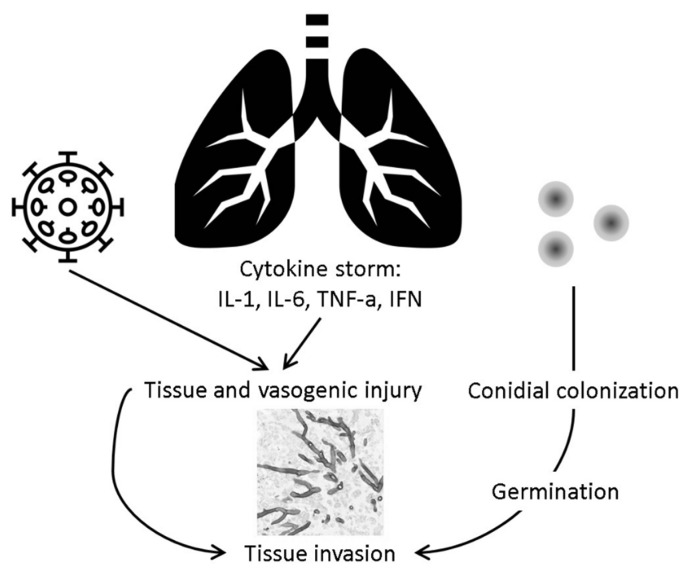
Proposed pathogenesis of CAPA.

The complex pathophysiology behind the development of CAPA is not well understood, but several hypotheses have been proposed (Figure 1). As with all infectious disease syndromes, the development of IPA in association with COVID-19 infection is a result of the interplay between host, pathogen, and environmental factors. *Aspergillus* spp. are abundant in the environment in the form of airborne conidia that can easily reach the alveoli. Alveolar macrophage-driven neutrophil recruitment and transient flux of the pro-inflammatory cytokine Tumor Necrosis Factor-α (TNF-α) are key factors in eliminating these conidia from the airways. Structural damage to the lungs by SARS-CoV-2 infection and an impaired immune system may provide adequate conditions for the conidia to germinate and produce hyphae that then invade the tissues and vessels.

Dexamethasone use has emerged as an important supportive treatment for COVID-19 [19]. Steroid use hampers the activity of alveolar macrophages and thereby facilitates the germination of conidia. Corticosteroids exert an anti-inflammatory effect by repression of the Nuclear Factor-kB (NF-kB)-dependent production of pro-inflammatory cytokines [20]. This was demonstrated in a mouse model of *Aspergillus* infection where mice treated with corticosteroids showed an absent TNF-α response [21]. The study also reported an elevated level of interleukin-10 (IL-10) with corticosteroid use. Elevated anti-inflammatory cytokines, such as IL-10, may favor IPA by inhibiting phagocytosis of *Aspergillus* spp. [22]. In addition, pathology analysis of neutropenic and glucocorticoid-treated mice models infected with intranasal *A. fumigatus* conidia, showed that, compared to neutropenia, corticosteroid-associated aspergillosis demonstrates extensive necrosis, less angioinvasion, and a lower fungal burden [22,23].

Notably, structural damage to the respiratory tissue caused by viral infections, such as influenza, may favor the development of concurrent fungal infections [24,25,26,27]. In moderate to severe COVID-19 infection, extensive damage to the lung parenchyma and vascular tissue can develop during the pro-inflammatory cytokine storm phase [28,29].

## 6. Clinical Presentation

COVID-19 initially presents with fever, dyspnea, cough, myalgia, and diarrhea, occasionally progressing to respiratory and multi-organ failure requiring mechanical ventilation and hemodynamic support [30,31,32]. In the majority of cases, a diagnosis of CAPA was established when respiratory status continued to worsen despite the management of ARDS. In addition to worsening respiratory failure, new, persistent, or rising fever can prompt further workup in some cases, leading to the diagnosis of CAPA. 

In one case, the question of secondary hemophagocytic lymphohistiocytosis (HLH) was raised due to elevated ferritin level (2442 ng/mL), decreased platelet count (83 × 10^9^/L) and hypofibrinogenemia (1.9 g/L). However, a diagnosis of HLH was not confirmed as these laboratory abnormalities may reflect the underlying pro-inflammatory state associated with COVID-19 [33]. Elevated ferritin level was reported in other studies as well (range: 465–2442 ng/mL) [34,35,36,37,38,39].

A total of 77 patients with CAPA (all with available data) required admission to the ICU, whereas this information was not available in one study [35]. Acute Respiratory Distress Syndrome (ARDS), which is the main complication associated with multi-organ failure in COVID-19, was reported in 21/85 cases (25%). Hemodynamic instability requiring vasopressor administration developed in 13/21 patients with ARDS (62%). Acute kidney injury developed in 20/21 patients with ARDS (95%), and nine (43%) required renal replacement therapy. No patients were receiving antifungal prophylaxis.

In contrast with influenza-associated acute pulmonary aspergillosis (IAPA, median time to onset three days), most cases presented late in the course of hospitalization, with a median time to diagnosis of 9 days (mean ± S.D 11.9 ± 10.6 days, range 0–53, interquartile range (IQR) 5–15, *n* = 42) from SARS-CoV-2 positivity (Table 1). 

## 7. Risk Factors

All 22 studies (*n* = 85 patients) reported patients’ comorbidities. The vast majority of patients (78/85, 91.8%) had no pre-existing immunocompromising or “immunomodulating” conditions. However, comorbidities such as type 2 diabetes mellitus, obesity, hypertension, COPD were fairly common (Table 1). 

Exceptions included (i) one patient who was a renal transplant recipient on maintenance immunosuppressive therapy (mycophenolate mofetil 1 g twice daily and prednisone 20 mg daily) [39], (ii) one patient who had a remote history of lung transplant on maintenance immunosuppressive therapy with tacrolimus and prednisone (unspecified doses) [40], (iii) one patient who was on chronic steroids for pemphigus foliaceus [9], (iv) one patient who had history of multiple myeloma and was on steroids [41], (v) one patient with history of rheumatoid arthritis on long-term leflunomide [33], (vi) one patient with a history of chronic myelomonocytic leukemia [40] and (vii) one patient with a history of unspecified hematologic malignancy [42]. For the last two patients, it was not specified in the studies whether their disease was active or in remission, and whether they were neutropenic at baseline or not. For the purposes of our review, we considered these five patients as immunocompromised hosts. 

There were three additional “immunomodulated” patients with history of hematologic malignancy for which they were not being treated. Two of these patients had been treated for acute myeloid leukemia (AML) remotely and were in remission [9,43]. The third was a patient with myelodysplastic syndrome (MDS) who was neither neutropenic nor on chemotherapy [44]. One other patient had a history of Human Immunodeficiency Virus (HIV) infection on lamivudine/tenofovir/nevirapine with a viral load of less than 20 copies and CD4 count > 250 cells [9]. We considered these patients as “immunomodulated”, non-immunocompromised hosts.

A total of 19 out of 21 studies reported information on corticosteroid use prior to the diagnosis of IPA. For one additional study, information regarding prior corticosteroid use was obtained after communication with the corresponding author, for a total of 72 patients [39]. Of these 72 patients, 38 patients (53%) had recently received systemic corticosteroids and one patient was on inhaled fluticasone for asthma prior to the diagnosis of IPA. Only four patients who received systemic corticosteroids had been on chronic steroids prior to diagnosis of COVID-19 (10%, patients (i)–(iv) above). The exact steroid regimens for patients (ii) and (iv) were not explicitly mentioned in the studies. All other patients in this subgroup (35/39, 90%) received corticosteroids at variable doses in the setting of their acute illness, either as an anti-inflammatory modality against severe COVID-19 or for refractory shock. A small number of patients received immunomodulators for the treatment of COVID-19 and prior to the diagnosis of IPA. Specifically, eight patients received tocilizumab [10,33,45,46] and one patient eculizumab [37]. 

## 8. Diagnosis 

**Figure 2:**
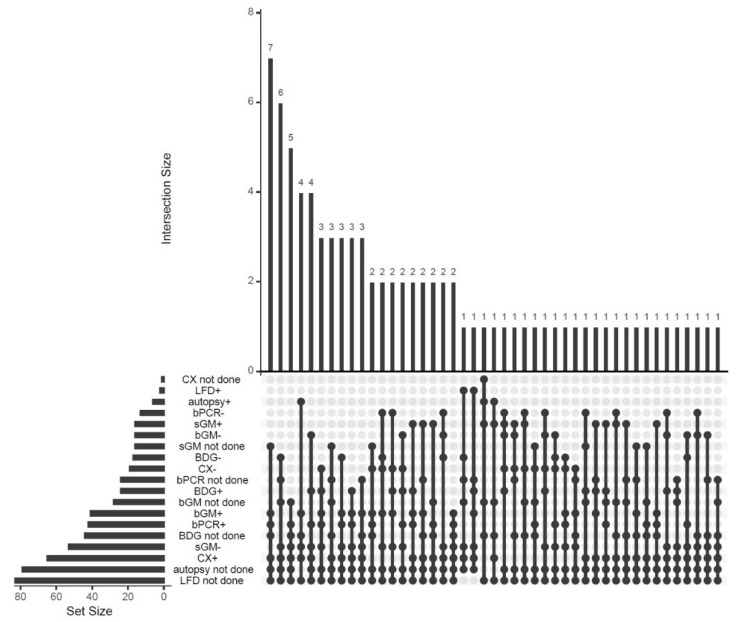
UpSet diagram of diagnostic modalities for the diagnosis of CAPA.

Diagnostic modalities included histopathologic examination of lung tissue on autopsy, recovery of *Aspergillus* sp. from tracheal aspirate, bronchial brush or bronchoalveolar lavage (BAL) culture (CX), *Aspergillus* sp. polymerase chain reaction (PCR) from a BAL sample, and fungal markers including serum galactomannan (sGM), BAL galactomannan (bGM) and serum beta-D-glucan (bDG). The following cut-offs were used for fungal marker positivity: sGM > 0.5, bGM > 1.0 and bDG > 7.0. Two case reports reported use of an *Aspergillus*-specific lateral flow device (LFD) test as an additional test for point-of-care diagnosis [34,35] (Figure 2). 

Amongst eighty-five cases of IPA, only six (7%) were proven by histopathology and all of them post-mortem, by autopsy. Forty-one (41, 48.2%) cases were classified as putative IPA, 26 (30.6%) as probable IPA (30.6%), and 12 (14.1%) as possible IPA. Respiratory tract cultures were performed in 84/85 patients and grew *Aspergillus* spp. in 65/85 (76.5%) cases. Aspergillus PCR from a BAL sample (bPCR) was performed in 61/85 patients and was positive in 42 of those 61 (68.8%) cases. bGM was performed in 57/85 patients and was positive in 41/57 (71.9%) cases. sGM was performed in 69/85 patients and was positive in 16/69 (23.2%) patients. Serum beta-D-glucan was performed in 41/85 patients and was positive in 25/41 (60.9%). Both patients in whom LFD was performed had a positive result. These results are summarized in Figure 2. 

The most frequent combination of tests was: 1. both positive culture and bPCR, and 2. bGM positive with neither sGM nor bDG performed (Figure 2).

## 9. Microbiology

Amongst the 85 cases of *Aspergillus* included in the studies, 67 were speciated (78.8%). In the case of one patient, both *A. fumigatus* and *A. flavus* were isolated from his BAL cultures, thus we analyzed 68 *Aspergillus* isolates [44]. The majority of *Aspergillus* isolates were identified as *A. fumigatus* (56/68, 82.3%), followed by *A. flavus* (8/68, 11.8%), *A. niger* (1/68, 1.5%), *A. terreus* (1/68, 1.5%), *A. tubingensis* (1/68, 1.5%) and *A. penicilloides* (1/68, 1.5%). 

There were three documented cases of multi-azole resistant strains, the first was a patient from a Dutch cohort [47] who had no known prior exposure to azoles but developed CAPA from *A. fumigatus* resistant to voriconazole, posaconazole and itraconazole. He was treated with intravenous (IV) liposomal amphotericin B (LAmB) and survived. 

The second was a patient from Ireland [43] who also had *A. fumigatus* resistant to voriconazole, posaconazole and itraconazole but this patient had both occupational exposure to fungicides and a past medical history of IPA in the setting of remote history of AML for which he had undergone chemotherapy. This patient also received IV LAmB but died. 

The last patient was treated in a tertiary center in the Netherlands [34] and had no known history of exposure to azoles. Her tracheal aspirate cultures upon ICU admission grew pan-sensitive *A. fumigatus* and the patient was initially treated with voriconazole. However, upon clinical deterioration two weeks later, cultures were repeated, and this time the isolated strain was resistant to itraconazole (minimal inhibitory concentration (MIC) 16 mg/L) and intermediate to voriconazole (MIC 2 mg/L) and posaconazole (MIC 0.5 mg/L), but sensitive to amphotericin B. Subsequently, the patient was switched from voriconazole to IV LAmB (LAmB); however, she did not survive. The authors reported detection of TR34/L98H mutation upon sequencing of the *A. fumigatus* cyp51A gene.

## 10. Treatment 

Seventy patients were treated. The most common treatment regimens were mold-active triazole (MAT) monotherapy (*n* = 38), echinocandin monotherapy (*n* = 2), (L)AmB monotherapy (*n* = 10), combination of mold-active azole and echinocandin (*n* = 9), combination of AmB and echinocandin (*n* = 1), combination of mold-active azole and AmB (*n* = 10). There was no significant association between antifungal treatment and all-cause mortality. Patients treated with voriconazole had a higher survival rate (53%, 29/55 vs. 33%, 10/30), although the difference did not reach statistical significance (*p* = 0.11). However, after adding the patients with CAPA from the Italian cohort [8], this signal was even stronger, 36/53 survivors were treated with voriconazole, compared to 32/62 who died (*p* = 0.089).

## 11. Outcomes

Case-fatality rate (CFR) was 54.1% (46/85), which is not substantially different from the high mortality reported in some studies with severe COVID-19 alone [30]. The only factor significantly associated with mortality was age. We also noted a trend for higher mortality in males, patients with diabetes, and those with history of asthma or chronic obstructive pulmonary disease (COPD) (Table 1). 

Three studies compared ICU CFR and outcomes between patients with CAPA and those with COVID-19 but no evidence of aspergillosis. Van Biesen et al., reported longer ICU stay with CAPA (37 days in CAPA vs. 19 days in non-CAPA, *p* < 0.05) without a difference in mortality (22.2% in those with CAPA vs. 15.1% in non-CAPA, *p* = 0.61) [39]; however, CAPA was diagnosed earlier in this study as part of active surveillance with serial fungal marker measurement. Alanio et al. observed similar mortality between CAPA and non-CAPA COVID-19 patients in the ICU (4/9, 44% vs. 7/18, 39%; *p* = 0.99) [41]. Van Arkel et al. found higher mortality in CAPA patients (4/6, 66.66% vs. 8/25, 32%); however, the difference was not significant (*p* = 0.174) [47]. 

Finally, we investigated if the SARS-CoV2 inoculum as quantified by RT-PCR cycle thresholds (Ct) could be associated with the severity of CAPA. However, only five studies (total of eight patients) referenced such data, and there was both specimen and gene target heterogeneity preventing further analysis. These data can be found in the Appendix A Table A1. 

## 12. Discussion

To our knowledge, this is the largest review and case series of patients with CAPA published in the English literature to-date. We highlight clinically relevant observations and uncharted areas that merit further investigation:

The vast majority of patients with CAPA did not fulfill classic host criteria, such as neutropenia, solid organ or hematopoetic cell transplantation (HCT), but rather had critical illness as their main predisposing factor. Likewise, in the prospective cohort of CAPA by Bartoletti et al., only a minority of patients met classic host criteria [8]. Furthermore, we demonstrated significant heterogeneity in laboratory modalities to diagnose CAPA, frequently based on >1 positive tests, more often bPCR and bGM (Figure 2). 

Although IPA in the ICU is an increasingly recognized syndrome, its diagnosis is often confounded by the possibility of colonization. It should be noted that the specificity of fungal biomarkers, especially bGM in patients without hematologic malignancies or history of HCT remains suboptimal, with very high false positive rates [48,49]. Therefore, it is possible that some patients diagnosed with CAPA may not have IPA. However, the absence of more classic hosts at risk for IPA from our case series, especially those with hematologic malignancies (HM), could be due to more strict infection control measures and neutropenic precautions in this patient population, as well as the frequent use of mold-active antifungal prophylaxis [50]. 

An important observation was that almost half of all patients with CAPA had corticosteroid exposure (Table 1). Steroids are increasingly recognized as an important risk factor for invasive mold infections [11,12,51,52], and more specifically mixed invasive mold infections [51,52], even in patients with no other risk factors [11,52]. Bartoletti et al., also pointed out that the high incidence of CAPA in their cohort could be associated with high utilization of corticosteroids and tocilizumab for COVID-19. Corticosteroids were associated with a statistically significant increase in mortality rates in their population (*p* = 0.04) [8]. Clinicians should have a low threshold for aggressive diagnostic work-up, and potentially early initiation of empiric antifungals in patients with COVID-19 receiving corticosteroids, especially as they are sometimes standard-of-care in critically-ill patients, based on the results of the RECOVERY randomized-controlled trial [19]. One major limitation of our systematic review is that many studies included did not report the specific dose and duration of corticosteroids, which are important for risk stratification.

We did not find a solid benefit from antifungal treatment, although the diagnosis of some cases post-mortem introduces by definition significant “immortal” bias favoring the treatment groups. Moreover, the only factors significantly associated with higher mortality rates were the same as those reported in the general population of patients with COVID-19, mainly age [29,52], but also comorbidities (diabetes, asthma, COPD) and, potentially, male sex [52,53]. Therefore, it is still not entirely clear if patients with CAPA have higher mortality than other ICU patients with COVID-19. However, the prospective study from Italy reported a significant association between bGM index at ICU admission and 30 day mortality (*p* = 0.014), after adjustment for other confounders [8]. 

The above points further emphasize the need for better differentiation between colonization and IPA, with accurate non-invasive methods, such as “breath tests” [54,55], since colonized-only patients are less likely to benefit from treatment. It should be noted, though, that, in our review, we captured a signal for mortality benefit in patients with voriconazole treatment, which almost reached statistical significance. Larger, prospective studies evaluating the effect of early antifungal treatment are needed. 

In conclusion, our findings indicate that the diagnosis of CAPA needs a strong correlation between clinical, radiographic and laboratory data [56,57,58,59,60], as the growth of *Aspergillus* or positive fungal biomarkers may not necessarily indicate invasive disease. High-risk patient groups need to be better defined, but administration of corticosteroids may be an important risk factor for CAPA. Clinical vigilance for CAPA is advisable in critically ill patients who are not improving, even those who do not meet classic host criteria. In such cases, a thorough, multi-faceted diagnostic work-up and early initiation of a MAT may be lifesaving.

## Figures and Tables

**Table 1:** Clinical characteristics, treatment and outcomes in patients with COVID-19-associated pulmonary aspergillosis (CAPA) who survived or died. Data are presented as N (%), unless otherwise indicated.

Parameter	Total	Survived	Died	*p*-Value
N	85	39	46	
Age (years, mean ± S.D.)	67.0 ± 11.5	63.2 ± 12.4	70.6 ± 9.5	0.0098
Missing/unknown	20 (24)	8 (21)	12 (26)	
Sex				
Female	16 (19)	11 (28)	5 (11)	0.083
Male	49 (58)	20 (51)	29 (63)	
Missing/unknown	20 (24)	8 (21)	12 (26)	
Comorbidities				
Asthma or chronic obstructive pulmonary disease (COPD)	17 (20)	4 (10)	13 (28)	0.056
Diabetes	15 (18)	4 (10)	11 (24)	0.15
Hypertension	20 (24)	9 (23)	11 (24)	>0.99
Corticosteroids	39 (46)	18 (46)	21 (46)	>0.99
Missing/unknown	13 (15)	6 (15)	7 (15)	
*Aspergillus* species *				
*A. fumigatus*	56 (66)	26 (67)	30 (65)	0.53
*A. flavus*	8 (9)	3 (8)	5 (11)	
*A. niger*	1 (1)	0 (0)	1 (2)	
*A. terreus*	1 (1)	0 (0)	1 (2)	
*A. penicilloides*	1 (1)	0 (0)	1 (2)	
*A. tubingensis*	1 (1)	1 (2)	0 (0)	
Unknown/not speciated	18 (21)	9 (23)	9 (20)	
Resistance	3 (4)	1 (3)	2 (4)	>0.99
Missing/unknown	69 (81)	34 (87)	35 (76)	
Time to diagnosis (days, median, interquartile range [IQR])	10, 5–15	12.5, 2.5–19.5	9, 6–13	0.86
Missing/unknown	43 (51)	20 (51)	23 (50)	
CAPA classification				
Proven	6 (7)	3 (8)	3 (7)	0.78
Probable or putative	67 (79)	31 (79)	36 (78)	
Possible	12 (14)	5 (13)	7 (15)	
Treatment **				
None	15 (18)	5 (13)	10 (22)	0.28
Mold-active triazole (MAT: vori-, posa-, isavuconazole)	61 (72)	31 (79)	30 (65)	0.61
Voriconazole	55 (65)	29 (74)	26 (57)	0.11
Isavuconazole	6 (7)	2 (5)	4 (9)	0.68
Echinocandin (caspo-, mica-, anidulafungin)	14 (16)	7 (18)	7 (15)	0.78
Amphotericin-B	21 (25)	11 (28)	10 (22)	0.62

* Total > 100% since one patient was coinfected with *A. fumigatus* and *A. flavus.* ** Total >100% since patients may have received combination or sequential antifungals.

## Appendix A

**Table A1:** SARS-CoV2 gene targets and cycle thresholds by real-time polymerase chair reaction (RT-PCR).

N	First Author	Specimen	RdRp Gene Ct	E Gene Ct	S Gene Ct	GADPH Gene Ct	ORF1 Gene Ct
1 *	Lescure [58]	NP, BAL	Negative	30.3, 27.4	Not done	25.7, 24.7	Not done
2	Ghelfelstein-Ferreira [37]	NP	Not done	23.3	22.8	Not done	Not done
3	Kohler [56]	BAL	Not done	13.29	12.61	Not done	Not done
4	Kohler [56]	BAL	Not done	34.29	Not done	Not done	31.47
5	Kohler [56]	BAL	Not done	29.74	Not done	Not done	27.86
6	Kohler [56]	TA	Not done	21.47	Not done	Not done	20.12
7	Meijer [34]	TA	Not done	30.59	Not done	Not done	Not done
8	Sharma [36]	NP	35.09	22.04	Not done	Not done	Not done

NP: nasopharyngeal, BAL: bronchoalveolar lavage, TA: tracheal aspirate, RdRp: RNA-dependent RNA polymerase, GADPH: Glyceraldehyde-3-phosphate dehydrogenase, ORF: open-reading frame, Ct: cycle threshold * Patient no. 1 had PCR performed from 2 different specimens on the same day.

## References

[1] (2020). WHO Statistics. https://www.who.int/docs/default-source/coronaviruse/situation-reports/20200726-covid-19-sitrep-188.pdf?sfvrsn=f177c3fa_2.

[2] Lansbury L., Lim B., Baskaran V., Lim W.S. (2020). Co-Infections in People with COVID-19: A Systematic Review and Meta-Analysis. SSRN Electron. J..

[3] De Pauw B., Walsh T.J., Donnelly J.P., Stevens D.A., Edwards J.E., Calandra T., Pappas P.G., Maertens J., Lortholary O., Kauffman C.A. (2008). Revised Definitions of Invasive Fungal Disease from the European Organization for Research and Treatment of Cancer/Invasive Fungal Infections Cooperative Group and the National Institute of Allergy and Infectious Diseases Mycoses Study Group (EORTC/MSG) Consensus Group. Clin. Infect. Dis..

[4] Blot S., Taccone F.S., Abeele A.-M.V.D., Bulpa P., Meersseman W., Brusselaers N., Dimopoulos G., Paiva J.A., Misset B., Rello J. (2012). A Clinical Algorithm to Diagnose Invasive Pulmonary Aspergillosis in Critically Ill Patients. Am. J. Respir. Crit. Care Med..

[5] Chen N., Zhou M., Dong X., Qu J., Gong F., Han Y., Qiu Y., Wang J., Liu Y., Wei Y. (2020). Epidemiological and clinical characteristics of 99 cases of 2019 novel coronavirus pneumonia in Wuhan, China: A descriptive study. Lancet.

[6] Wang J., Yang Q., Zhang P., Sheng J., Zhou J., Qu T. (2020). Clinical characteristics of invasive pulmonary aspergillosis in patients with COVID-19 in Zhejiang, China: A retrospective case series. Crit. Care.

[7] Flikweert A.W., Grootenboers M.J., Yick D.C., Du Mée A.W., Van Der Meer N.J., Rettig T.C., Kant M.K. (2020). Late histopathologic characteristics of critically ill COVID-19 patients: Different phenotypes without evidence of invasive aspergillosis, a case series. J. Crit. Care.

[8] Bartoletti M., Pascale R., Cricca M., Rinaldi M., Maccaro A., Bussini L., Fornaro G., Tonetti T., Pizzilli G., Francalanci E. (2020). Epidemiology of Invasive Pulmonary Aspergillosis Among Intubated Patients With COVID-19: A Prospective Study. Clin. Infect. Dis..

[9] Rutsaert L., Steinfort N., Van Hunsel T., Bomans P., Naesens R., Mertes H., Dits H., Van Regenmortel N. (2020). COVID-19-associated invasive pulmonary aspergillosis. Ann. Intensiv. Care.

[10] Lamoth F., Glampedakis E., Boillat-Blanco N., Oddo M., Pagani J.-L. (2020). Incidence of invasive pulmonary aspergillosis among critically ill COVID-19 patients. Clin. Microbiol. Infect..

[11] Lionakis M.S., Kontoyiannis D.P. (2003). Glucocorticoids and invasive fungal infections. Lancet.

[12] Kitmiridou D., Aung S., Farmakiotis D. (2018). Disseminated Mucormycosis with Positive Aspergillus Galactomannan. Case Rep. Infect. Dis..

[13] Meersseman W., Lagrou K., Maertens J., Van Wijngaerden E. (2007). Invasive Aspergillosis in the Intensive Care Unit. Clin. Infect. Dis..

[14] Schauwvlieghe A.F.A.D., Rijnders B., Philips N., Verwijs R., Vanderbeke L., Van Tienen C., Lagrou K., Verweij P.E., Van De Veerdonk F.L., Gommers D. (2018). Invasive aspergillosis in patients admitted to the intensive care unit with severe influenza: A retrospective cohort study. Lancet Respir. Med..

[15] Van De Veerdonk F., Kolwijck E., Lestrade P.P.A., Hodiamont C.J., Rijnders B., Van Paassen J., Haas P.-J.A., Dos Santos C.O., Kampinga G.A., Bergmans D.C.J.J. (2017). Influenza-associated Aspergillosis in Critically Ill Patients. Am. J. Respir. Crit. Care Med..

[16] Latgé J.-P. (2001). The pathobiology of Aspergillus fumigatus. Trends Microbiol..

[17] Latgé J.-P., Chamilos G. (2019). Aspergillus fumigatus and Aspergillosis in 2019. Clin. Microbiol. Rev..

[18] Latgé J.-P. (1999). Aspergillus fumigatus and Aspergillosis. Clin. Microbiol. Rev..

[19] Horby P., Lim W.S., Emberson J.R., Mafham M., Bell J.L., Linsell L., Staplin N., Brightling C., Ustianowski A., RECOVERY Collaborative Group (2020). Dexamethasone in Hospitalized Patients with Covid-19—Preliminary Report. N. Engl. J. Med..

[20] Yamamoto Y., Gaynor R.B. (2001). Therapeutic potential of inhibition of the NF-kappaB pathway in the treatment of inflammation and cancer. J. Clin. Investig..

[21] Balloy V., Huerre M., LatgéJ -P., Chignard M. (2005). Differences in Patterns of Infection and Inflammation for Corticosteroid Treatment and Chemotherapy in Experimental Invasive Pulmonary Aspergillosis. Infect. Immun..

[22] Roilides E., Sein T., Roden M., Schaufele R.L., Walsh T.J. (2001). Elevated Serum Concentrations of Interleukin-10 in Nonneutropenic Patients with Invasive Aspergillosis. J. Infect. Dis..

[23] Lewis R.E., Kontoyiannis D.P. (2009). Invasive aspergillosis in glucocorticoid-treated patients. Med. Mycol..

[24] Vanderbeke L., Spriet I., Breynaert C., Rijnders B.J., Verweij P.E., Wauters J. (2018). Invasive pulmonary aspergillosis complicating severe influenza. Curr. Opin. Infect. Dis..

[25] Bautista E., Chotpitayasunondh T., Gao Z., Harper S.A., Shaw M., Uyeki T.M., Zaki S.R., Hayden F.G., Hui D.S., Writing Committee of the WHO Consultation on Clinical Aspects of Pandemic (H1N1) 2009 Influenza (2010). Clinical Aspects of Pandemic 2009 Influenza A (H1N1) Virus Infection. N. Engl. J. Med..

[26] Ajmal S., Mahmood M., Abu Saleh O., Larson J., Sohail M. (2018). Invasive fungal infections associated with prior respiratory viral infections in immunocompromised hosts. Infection.

[27] Crum-Cianflone N. (2016). Invasive Aspergillosis Associated With Severe Influenza Infections. Open Forum Infect. Dis..

[28] Schaller T., Hirschbühl K., Burkhardt K., Braun G., Trepel M., Märkl B., Claus R. (2020). Postmortem Examination of Patients With COVID-19. JAMA.

[29] Menter T., Haslbauer J.D., Nienhold R., Savic S., Hopfer H., Deigendesch N., Frank S., Turek D., Willi N., Pargger H. (2020). Post-mortem examination of COVID19 patients reveals diffuse alveolar damage with severe capillary congestion and variegated findings of lungs and other organs suggesting vascular dysfunction. Histopathology.

[30] Yang X., Yu Y., Xu J., Shu H., Xia J., Liu H., Wu Y., Zhang L., Yu Z., Fang M. (2020). Clinical course and outcomes of critically ill patients with SARS-CoV-2 pneumonia in Wuhan, China: A single-centered, retrospective, observational study. Lancet Respir. Med..

[31] Gandhi R.T., Lynch J.B., Del Rio C. (2020). Mild or Moderate Covid-19. N. Engl. J. Med..

[32] Berlin D.A., Gulick R.M., Martinez F.J. (2020). Severe Covid-19. N. Engl. J. Med..

[33] Cai S., Sun W., Li M., Dong L. (2020). A complex COVID-19 case with rheumatoid arthritis treated with tocilizumab. Clin. Rheumatol..

[34] Meijer E.F.J., Dofferhoff A.S., Hoiting O., Buil J.B., Meis J.F. (2020). Azole-Resistant COVID-19-Associated Pulmonary Aspergillosis in an Immunocompetent Host: A Case Report. J. Fungi.

[35] Prattes J., Valentin T., Hoenigl M., Talakic E., Reisinger A.C., Eller P. (2020). Invasive pulmonary aspergillosis complicating COVID-19 in the ICU—A case report. Med. Mycol. Case Rep..

[36] Sharma A., Hofmeyr A., Bansal A., Thakkar D., Lam L., Harrington Z., Bhonagiri D. (2020). COVID-19 associated pulmonary aspergillosis (CAPA): An Australian case report. Med. Mycol. Case Rep..

[37] Ghelfenstein-Ferreira T., Saade A., Alanio A., Bretagne S., De Castro R.J.A., Hamane S., Azoulay E., Bredin S., Dellière S. (2020). Recovery of a triazole-resistant Aspergillus fumigatus in respiratory specimen of COVID-19 patient in ICU—A case report. Med. Mycol. Case Rep..

[38] Fernandez N.B., Caceres D.H., Beer K.D., Irrazabal C., Delgado G., Farias L., Chiller T.M., Verweij P.E., Stecher D. (2020). Ventilator-associated pneumonia involving Aspergillus flavus in a patient with coronavirus disease 2019 (COVID-19) from Argentina. Med. Mycol. Case Rep..

[39] Van Biesen S., Kwa D., Bosman R.J., Juffermans N.P. (2020). Detection of Invasive Pulmonary Aspergillosis in COVID-19 with Non-directed Bronchoalveolar Lavage. Am. J. Respir. Crit. Care Med..

[40] Gangneux J.-P., Bougnoux M.-E., Dannaoui E., Cornet M., Zahar J., Ralph Z.J. (2020). Invasive fungal diseases during COVID-19: We should be prepared. J. Mycol. Med..

[41] Alanio A., Dellière S., Fodil S., Bretagne S., Mégarbane B. (2020). Prevalence of putative invasive pulmonary aspergillosis in critically ill patients with COVID-19. Lancet Respir. Med..

[42] White L., Dhillon R., Cordey A., Hughes H., Faggian F., Soni S., Pandey M., Whitaker H., May A., Morgan M. (2020). A National Strategy to Diagnose COVID-19 Associated Invasive Fungal Disease in the ICU. SSRN Electron. J..

[43] Mohamed A., Rogers T.R., Talento A.F. (2020). COVID-19 Associated Invasive Pulmonary Aspergillosis: Diagnostic and Therapeutic Challenges. J. Fungi.

[44] Blaize M., Mayaux J., Nabet C., Lampros A., Marcelin A.-G., Thellier M., Piarroux R., Demoule A., Fekkar A. (2020). Fatal Invasive Aspergillosis and Coronavirus Disease in an Immunocompetent Patient. Emerg. Infect. Dis..

[45] Nasir N., Farooqi J.Q., Mahmood S.F., Jabeen K. (2020). COVID-19-associated pulmonary aspergillosis (CAPA) in patients admitted with severe COVID-19 pneumonia: An observational study from Pakistan. Mycoses.

[46] Bruno G., Fabrizio C., Buccoliero G.B. (2020). COVID-19-associated pulmonary aspergillosis: Adding insult to injury. Lancet Microbe.

[47] Brown L.-A.K., Ellis J., Gorton R., De S., Stone N. (2020). Surveillance for COVID-19-associated pulmonary aspergillosis. Lancet Microbe.

[48] Farmakiotis D., Le A., Weiss Z.F., Ismail N., Kubiak D.W., Koo S. (2018). False positive bronchoalveolar lavage galactomannan: Effect of host and cut-off value. Mycoses.

[49] Affolter K., Tamm M., Jahn K., Halter J., Passweg J.R., Hirsch H.H., Stolz D. (2014). Galactomannan in the Bronchoalveolar Lavage for Diagnosing Invasive Fungal Disease. Am. J. Respir. Crit. Care Med..

[50] Hsu A., Matera R., Vieira K., Reagan J.L., Farmakiotis D. (2020). Antifungal prophylaxis during 7 + 3 induction chemotherapy for acute myeloid leukemia is associated with improved survival, in a setting with low incidence of invasive mold infections. Support. Care Cancer.

[51] Tsikala-Vafea M., Cao W., Olszewski A.J., Donahue J.E., Farmakiotis D. (2020). Fatal Mucormycosis and Aspergillosis in an Atypical Host: What Do We Know about Mixed Invasive Mold Infections?. Case Rep. Infect. Dis..

[52] Magira E.E., Jiang Y., Economides M., Tarrand J., Kontoyiannis D.P. (2018). Mixed mold pulmonary infections in haematological cancer patients in a tertiary care cancer centre. Mycoses.

[53] Kuderer N.M., Choueiri T.K., Shah D.P., Shyr Y., Rubinstein S.M., Rivera D.R., Shete S., Hsu C.-Y., Desai A., Lopes G.D.L. (2020). Clinical impact of COVID-19 on patients with cancer (CCC19): A cohort study. Lancet.

[54] Acharige M.J.T., Koshy S., Ismail N., Aloum O., Jazaerly M., Astudillo C.L., Koo S. (2018). Breath-based diagnosis of fungal infections. J. Breath Res..

[55] Koo S., Thomas H.R., Daniels S.D., Lynch R.C., Fortier S.M., Shea M.M., Rearden P., Comolli J.C., Baden L.R., Marty F.M. (2014). A Breath Fungal Secondary Metabolite Signature to Diagnose Invasive Aspergillosis. Clin. Infect. Dis..

[56] Koehler P., Cornely O.A., Böttiger B.W., Dusse F., Eichenauer D.A., Fuchs F., Hallek M., Jung N., Klein F., Persigehl T. (2020). COVID-19 associated pulmonary aspergillosis. Mycoses.

[57] Santana M.F., Pivoto G., Alexandre M.A.A., Baía-Da-Silva D.C., Borba M.G.D.S., Val F.A., Brito-Sousa J.D., Melo G.C., Monteiro W.M., Souza J.V.B. (2020). Confirmed Invasive Pulmonary Aspergillosis and COVID-19: The value of postmortem findings to support antemortem management. Rev. Soc. Bras. Med. Trop..

[58] Lescure F.-X., Bouadma L., Nguyen D., Parisey M., Wicky P.-H., Behillil S., Gaymard A., Bouscambert-Duchamp M., Donati F., Le Hingrat Q. (2020). Clinical and virological data of the first cases of COVID-19 in Europe: A case series. Lancet Infect. Dis..

[59] Lahmer T., Rasch S., Spinner C., Geisler F., Schmid R., Huber W. (2020). Invasive pulmonary aspergillosis in severe coronavirus disease 2019 pneumonia. Clin. Microbiol. Infect..

[60] Antinori S., Rech R., Galimberti L., Castelli A., Angeli E., Fossali T., Bernasconi D., Covizzi A., Bonazzetti C., Torre A. (2020). Invasive pulmonary aspergillosis complicating SARS-CoV-2 pneumonia: A diagnostic challenge. Travel Med. Infect. Dis..

